# Photobiomodulation and Pain Reduction in Patients Requiring Orthodontic Band Application: Randomized Clinical Trial

**DOI:** 10.1155/2020/7460938

**Published:** 2020-05-25

**Authors:** Maria Francesca Sfondrini, Marina Vitale, Antonio Luiz Barbosa Pinheiro, Paola Gandini, Lorenzo Sorrentino, Ugo Matteo Iarussi, Andrea Scribante

**Affiliations:** ^1^Unit of Orthodontics and Paediatric Dentistry, Section of Dentistry-Department of Clinical, Surgical, Diagnostic and Paediatric Sciences, University of Pavia, Pavia, Italy; ^2^Center of Biophotonics, Dental School, Federal University of Bahia (UFBA), Salvador, BA, Brazil

## Abstract

**Purpose:**

The aim of this study was to investigate the effect of Photobiomodulation (PBM) in managing orthodontic pain intensity over time in patients requiring band application on upper first molars.

**Methods:**

Maxillary first molars were banded. In the trial group, each molar received single-session PBM on two buccal and two palatal points (*λ* = 830 ± 10 nm; 150 mW, 7.5 J/cm^2^; spot of 0.1 cm^2^; 5 sec per point), while the control group received a placebo treatment. All patients were asked to answer five pain rating scales to assess pain intensity at 5 minutes and 1, 12, 24, and 72 hours and completed a survey describing the type of pain and its temporal course in the next 7 days.

**Results:**

26 patients (mean age 11.8 years) were randomly assigned to a control or a trial group. The trial group showed significantly lower pain intensities (*p* < 0.05) at 5 min (*M* = 0.92, SD = 1.32), 1 h (*M* = 0.77, SD = 1.01), and 12 h (*M* = 0.77, SD = 1.54) after band application compared to the control group (5 min: *M* = 1.62, SD = 1.26; 1 h: *M* = 1.77, SD = 1.92; and 12 h: *M* = 1.77, SD = 2.17), whereas no difference between groups (*p* > 0.05) was found at 24 h (trial: *M* = 0.62, SD = 1.71; control: *M* = 1.08, SD = 1.75) and 72 h (trial: *M* = 0.31, SD = 0.75; control: *M* = 0.15, SD = 0.55). Patients in the control group reported more frequently the presence of “compressive pain” (58.8%, *p* < 0.05) from the appliance during the week after the application, while the trial group showed higher frequency of “no pain” (46.2%, *p* < 0.05). However, PBM did not affect the pain onset (trial: *M* = 10.86, SD = 26.97; control: *M* = 5.25, SD = 7.86), peak (trial: *M* = 15.86, SD = 26.29; control: 6.17, SD = 7.96), and end time (trial: 39.57, SD = 31.33; control: *M* = 22.02, SD = 25.42) reported by the two groups (*p* > 0.05).

**Conclusions:**

PBM might be considered a promising alternative to decrease general pain intensity, although not affecting the typical pain cycle, in terms of the onset, peak, and ending times.

## 1. Introduction

Pain is an unpleasant sensory and emotional experience associated with actual or potential tissue damage [1], and it is one of the most frequent complications of orthodontics that might reduce patient compliance and treatment withdrawal [2, 3]. The orthodontic forces promote tooth displacement in the periodontal ligament space, leading to bone remodeling of the alveolus through bone resorption and apposition [4]. These processes most often result in pain [3], since they are related to reactions such as changes in blood flow, release of inflammatory cytokines (histamine, prostaglandins, encephalin, substance P, leukotrienes, etc.), stimulation of afferent A delta and C nerve fibers, release of neuropeptides, and hyperalgesia [5, 6]. Several studies evaluated the effectiveness of different methodologies to control orthodontic pain. In this regard, Nonsteroidal Anti-Inflammatory Drugs (NSAIDs) are one of the most common and effective ways to manage orthodontic pain by means of cyclooxygenase enzyme system inhibition, associated with decreased prostaglandin synthesis [7, 8]. Nevertheless, many side effects like allergies, gastric ulcers, bleeding disorders [8, 9], and reduction of tooth movement rate have been reported [9]. Interestingly, several alternatives have been investigated with the aim of reducing pain such as vibrational devices, cognitive and music therapy, muscle relaxation, or other psychological interventions [10, 11]. However, clinical application of these alternatives has been limited due to unclear results and lacking strong evidence.

Lasers have been introduced in dentistry for many clinical procedures: diagnosis [12], oral surgery [13], cavity preparation [14], and enamel conditioning [15]. Additionally, lasers have a great importance in combined surgical/orthodontic procedures, such as exposure of partially erupted teeth [16], thus reducing blood contamination that would negatively influence appliance efficiency [17, 18]. Finally, lasers can be applied to obtain photobiomodulation (PBM) [19]. Critically, PBM therapy, also known as Low-Level Laser Therapy (LLLT), has been shown to reduce pain in various fields of dentistry, including orthodontics [8, 20–27]. Hence, attention on this therapy is increasing due to its advantages in analgesia, biostimulation, and limited adverse effects [28–31]. Although the underlying mechanism requires further investigations, PBM analgesic action has been explained by stimulation of nerve cells, stabilization of membrane potentials, and release of neurotransmitters in the inflammatory tissue [32–34]. In particular, it has also been postulated that the effects of PBM on pain attenuation can be attributed to different aspects: inhibitory effects on nerve depolarization (especially C fibers), production of energy molecules (ATP), and reduction of prostaglandin levels [30, 35–37]. In orthodontics, PBM analgesic effect has generally been applied for postadjustment pain reduction and has been found to diminish orthodontic pain [7, 9, 10].

The present study was aimed at evaluating the effect of PBM on pain in patients undergoing band application on maxillary first molars for orthodontic treatment. Indeed, metal band insertion, especially in the case of tight dental contacts, might lead to pressure sensation, bite sensitivity, and pain to banded teeth [38, 39], due to interproximal space expansion and associated orthodontic force application. Moreover, pain during orthodontic treatment usually starts two hours after the application of orthodontic force, reaches a peak level at 24 hours, and lasts approximately five days [8, 38, 40, 41]. In this regard, the present study also assessed whether PBM might modulate the typical temporal course of orthodontic pain. The objective of the study was to estimate the efficacy over time of PBM compared to the untreated control group after orthodontic band cementation. The null hypothesis was that there is no significant difference between trial and control groups in pain measurements and questionnaire results.

## 2. Materials and Methods

**Figure 1:**
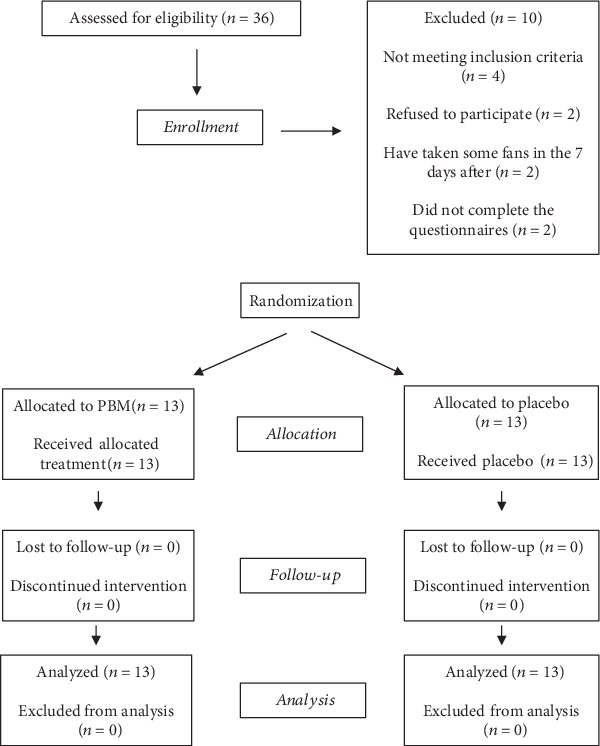
Participants' flow.

Figure 1 shows the participant flow of patients through the trial. In the present study, there were neither losses nor exclusions after randomization.

Baseline characteristics of the patients in the two groups are reported in Table 1. Twenty-six orthodontic patients (9 M and 17 F), aged between 7.2 and 20 years (mean age = 11.8, SD = 3.6), participated in the study. Half of the sample (*N* = 13; 5 M and 8 F), aged between 7 and 19 years (mean age = 11.7, SD = 3.7), was assigned to the control group, and the other half (*N* = 13; 4 M and 9 F), aged between 7 and 20 years (mean age = 11.9, SD = 3.7), was assigned to the trial group. Written informed consent was obtained from all patients (or patients' parents/legal tutors for participants younger than 18 years old).

The following selection criteria were applied:

- Presence of mixed or permanent dentition
- Need for orthodontic bands on upper first molars
- Fully erupted upper first molars
- Absence of pathological conditions associated with teeth, gingiva, or periodontium and good oral hygiene (gingival index < 1, plaque index ≤ 1, probing pocket depth ≤ 3 mm, and no Clinical Attachment Level (CAL) loss = 0)
- Absence of systemic, neurological, or psychiatric disorders
- Absence of chronic pain
- No systemic medication assumption three days before
- Absence of oral surgery interventions in the 30 days before the procedure
- Absence of melanin gingival pigmentations, restorations, or spaces adjacent to the site where the orthodontic bands need to be inserted
- Absence of posterior crowding (no need for elastomeric separators)

Patients who met the selection criteria were prospectively recruited from the Unit of Orthodontics and Paediatric Dentistry, Section of Dentistry, Department of Clinical, Surgical, Diagnostic and Paediatric Sciences, University of Pavia, Italy. Participants were recruited from 30/01/2019 to 29/10/2019.

### 2.1. Experimental Design

The present study was a parallel-group, single-blinded, placebo-controlled, randomized clinical trial with a 1 : 1 allocation ratio.

The Internal Review Board approved the study design.

No changes to the methods after trial commencement occurred.

A single examiner (LS) selected patients by administering an anamnestique questionnaire and performing the clinical evaluation to ensure that all subjects fulfilled all the inclusion criteria.

A power analysis based on previous studies [22, 42] indicated that a sample size of 13 subjects per group would be required to have 80% power at *p* < 0.05. Therefore, 13 subjects were enrolled per group.

Randomized sequence was generated with computer software (R® version 3.1.3, R Development Core Team, R Foundation for Statistical Computing, Wien, Austria).

In order to ensure equal distribution, all eligible patients were randomly allocated in either the control or the trial group by means of the randomization table.

For allocation concealment, the operator that applied the bands (UMR) was not aware of the allocation group of the patients. The operator in charge of the laser or placebo (LS) consulted the randomization list and performed the corresponding procedure. The randomization list was generated and held securely in a remote location.

For the implementation of the randomization, the allocation sequence was generated by the first operator (MFS). She was blinded to clinical visits and measures. The discussion with the patients explaining study design was performed by an operator (PG) that was blinded to both clinical measures and randomization list generation. Another clinician (LS) enrolled participants and assigned them to the corresponding group of intervention (laser vs. control) following the randomization list.

The patients were blind to their type of intervention. Blinding of the operator who performed the procedure (LS) was not possible.

### 2.2. Band Placement

A clinician (UMR) from the Unit of Orthodontics cemented two bands for each participant (3M, Unitek Molar Bands, Saint Paul, USA) on upper first molars for a multiband-multibracket orthodontic treatment.

Since posterior crowding and closeness to the adjacent teeth might cause painful band insertion and require the application of elastomeric separators [43], we included only patients with the absence of posterior crowding and second molars not fully erupted, thus ensuring lack of tight contacts.

### 2.3. Laser Application

After band placement, participants in the trial group received one session of PBM therapy, whereas participants in the control group did not receive any laser application for pain control.

**Figure 2:**
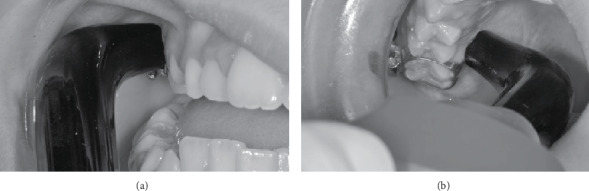
Irradiation points of buccal (a) and palatal sites (b).

The device used in this study was a diode laser, GaAlAs (Ultra Blue IV Plus, DMC Equipamentos, São Carlos, Brasile; *λ* = 830 ± 10 nm, 150 mW, 5 s, 7.5 J/cm^2^, spot of 0.1 cm^2^) (Table 2). After mucosa drying with an air flow, the laser was applied in 4 points for each banded molar (2 mm apically from the gingival margin): on the mesiobuccal (MB), distobuccal (DB), mesiopalatal (MP), and distopalatal (DP) portions of the teeth (see Figure 2). The device power was 500 mW, as declared by the producing company; however, the real power output at the tip was 150 ± 10 mW as measured by the Physics Department of the University of Pavia through the Ophir power meter (Ophir Photonics, Jerusalem, Israel). The exposure time for each point was 5 seconds. Laser-related experimental procedures were performed by the same operator (LS).

Participants in both groups were asked to avoid the use of any drugs during the experimental period (one week); otherwise, they were excluded from the study.

According to biosafety rules, the patients and the operator wore safety glasses during laser irradiation and simulated laser application (control group). For the control group, to ensure patient blinding, a silicon block was added between the probe and the laser source in order to prevent laser emission while recreating the same acoustic sensations of the real laser intervention.

The main outcome of the present study was the assessment of pain intensity reduction induced by PBM as compared to a placebo treatment. The secondary outcome was the evaluation of the PBM protocol on the typical course of orthodontic pain during time and on the type of pain through a four-question inventory.

**Figure 3:**
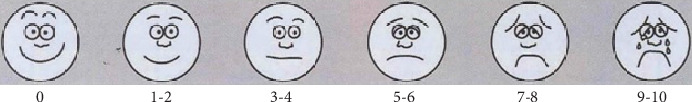
The Wong-Baker faces pain rating scale used in the present report, with the corresponding values of the conventional VAS scores.

After the intervention, to measure both primary and secondary outcomes, participants were asked to answer five Wong-Backer faces pain rating scales (WBS) to assess pain intensity at different time points: 5 minutes after band application (T0), after 1 hour (T1), after 12 hours (T2), after 24 hours (T3), and after 72 hours (T4). WBS is a paediatric adaptation to conventional visual analogue scale (VAS) conventionally used to evaluate a patient's comfort [44]. The WBS has been used since it has been demonstrated to be a validated measure for pain assessment in pediatric patients [45, 46], and it has been used to evaluate the efficacy of PBM on orthodontic pain [47, 48] (Figure 3). Moreover, a week after the intervention, to investigate the secondary outcome, patients completed a modified version of the Harazaki questionnaire [49, 50], composed of four questions: (Q1) “How many hours after the intervention did the pain start?”; (Q2) “When did you have the most serious pain?”; (Q3) “When did the pain disappear?”; and finally, (Q4) patients were asked to record their type of pain according to five categories of oral symptoms: (1) no pain, (2) compressive pain from the appliance, (3) pain when biting firmly but without eating problems, (4) pain when eating, and (5) spontaneous pain or pain which prevents eating. Participants who did not complete all questionnaires and rating scales were excluded from the analysis and considered dropouts.

There were no outcome changes after trial commencement.

### 2.4. Statistical Analysis

Statistical analysis was performed with R software (R version 3.1.3, R Development Core Team, R Foundation for Statistical Computing, Wien, Austria). Descriptive statistics, including the mean, standard deviation, median, and minimum and maximum values, were calculated for all groups.

The normality of the data was calculated using the Kolmogorov-Smirnov test. Analysis of variance (ANOVA) was applied to determine whether significant differences in WBS values existed among the various groups. The Tukey test was assessed post hoc.

Concerning the results of questions Q1, Q2, and Q3, a *t*-test was applied for each variable. Results of Q4 were analyzed with the chi-squared test.

Significance for all statistical tests was predetermined at *p* < 0.05.

## 3. Results

**Figure 4:**
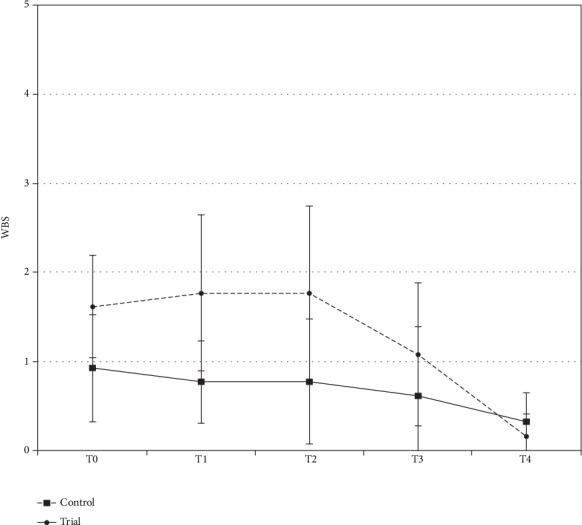
WBS values (mean and CI) of the two groups.

Descriptive statistics are presented in Table 3. ANOVA showed the presence of significant differences between the two groups (*p* < 0.01). As shown in Figure 4, post hoc Tukey testing showed that the control group showed the highest WBS values (*p* < 0.05) at T0, T1, and T2, with no significant differences among the three observation times (*p* > 0.05). WBS scores decreased at T3 and exhibited significantly the lowest values at T4.

Concerning the trial group, the highest WBS values (*p* < 0.05) were shown at T0, T1, and T2, with no significant differences among the three observation times (*p* > 0.05). WBS scores decreased at T3 and exhibited significantly the lowest values at T4.

Additionally, the control group exhibited significantly higher WBS scores than the trial group at T0, T1, and T2 (*p* < 0.05). No significant differences between the two groups were reported at T3 and T4 (*p* > 0.05).

Concerning the four questions (Table 4), Q1, Q2, and Q3 exhibited no significant differences between the control and trial groups (*p* > 0.05). On the other hand, Q4 showed a significant higher score of “2” (compressive pain from the appliance) for the control group and a greater frequency of score “1” (no pain) for the trial group (*p* < 0.05) (Table 5).

In the present study, no harm was reported.

## 4. Discussion

In the present study, pain intensity induced by band application on upper first molars was measured through WBS, after 5 minutes, 1 h, and 12 h to test the acute effect of PBM on instant pain, our primary outcome. Subsequently, the same measures were collected after 24 and 72 h to cover the peak period. Moreover, a modified version of the Harazaki and Isshiki questionnaire [49], in line with prior evidence [41, 50], was also submitted to investigate the type of pain, when it started, peaked, and disappeared, which was our secondary outcome.

The null hypothesis (that is, no significant difference between trial and control groups in pain measurements and questionnaire results) was partially rejected.

The results about the primary outcome showed that PBM is able to reduce pain intensity induced by band application on upper first molars, as shown by lower WBS values for the trial group as compared to those for the control group.

As regards the secondary outcome, the results demonstrated that PBM is able to reduce pain intensity during the first 12 hours after band insertion. Indeed, the trial group showed lower WBS values 5 minutes, 1 h, and 12 h after force application, whereas no difference between groups was found in the follow-up assessments (24 and 72 h after the orthodontic procedure). Critically, PBM did not affect the typical pain temporal course, as demonstrated by the comparable onset, peak, and end time of pain reported by the two groups in Q1, Q2, and Q3. Accordingly, in both groups, the highest WBS values were shown at T0, T1, and T2, with no differences among the three observations; WBS scores decreased at T3 and exhibited the lowest values at T4. Therefore, the lack of difference in WBS values between groups in T3 and T4 might be attributable to the physiological decrease in pain in the control group after the first 12 hours, which leads to comparable values between groups. Concerning the evaluation of pain type (Q4), the control group more frequently reported the presence of “compressive pain from the appliance,” while the trial group reported “no pain” with a higher frequency. Hence, PBM therapy might be considered a promising alternative to decrease general pain intensity, although not affecting the typical pain cycle, in terms of the onset, peak, and ending times.

PBM has been recently proposed as a valuable treatment for orthodontic pain due to its advantages in analgesia, biostimulation, and limited adverse effects [28–31]. The efficacy of PBM therapy can be influenced by different factors including light source, power output, wavelength, spot size, energy density, mode of operation (continuous or pulsed wave), time of exposure, application interval, and frequency [51, 52]. PBM therapy has been demonstrated to be effective in pain management in several orthodontic procedures, like separator placement [6, 41, 53], canine retraction [25, 54], and both initial [50, 55] and final stages [56] of archwire placement. To date, only one study [38] investigated PBM efficacy in reduction of pain sensations caused by molar band placement. However, a direct comparison with this study was not possible because of the different methodology adopted in terms of study design (split-mouth vs. placebo-controlled), number of laser sessions (two vs. one), and time points chosen to measure pain intensity. A placebo-controlled study design was adopted for this clinical trial, since it has been previously demonstrated that PBM might induce effects on the central nervous system with a systemic effect, through ascending and descending transmission modulation [37, 57], with effects possibly spreading to the nontreated half mouth. Nevertheless, there is no consensus in the literature about the best study design for PBM clinical trials, whether split-mouth [20, 22, 52, 53] or placebo-controlled [41, 50, 51].

It has been demonstrated that orthodontic pain generally starts at 2 h and peaks at 12-24 h after initial force application [38, 41]; however, severe pain may occur immediately if an acute and heavy force, such as a separator or a band, is applied on teeth [38, 58].

The results of the present study are in line with previous findings showing the efficacy of PBM in different orthodontic procedures [38, 53, 55, 59]; nevertheless, other studies did not report similar results [20, 26, 42]. Controversial evidence has been also reported for the PBM effect on the pain cycle. Indeed, although several studies, in line with the present one, failed to report a PBM-induced modulation of the typical pain time course [40, 50], other evidence reported opposite results, suggesting that PBM might be able to shift forward the peak time [49, 51] and anticipate the end of pain [41, 52]. It is important to note that such discrepant results might be related to the great variation in study designs and laser parameters among previous researches. In the present study, a laser with a wavelength of 830 nm has been used, since it has been previously demonstrated to have the efficacy of wavelengths ranging from 670 nm to 830 nm [38, 53]. Accordingly, wavelength lower than 600 nm would be absorbed by hemoglobin, whereas those above 1150 nm would be absorbed by water in tissues [19, 60]. Moreover, an energy density of 7.5 J/cm^2^ was chosen, because PBM seems to require an energy density between 0.05 and 10 J/cm^2^: in fact, values greater than 10 J/cm^2^ can lead to a bioinhibitory effect [19, 60]. Critically, although previous data reported that the biomodulatory effect seems greater for exposure times from 30 to 120 sec [19, 20, 22], the data of the present study confirmed prior evidence [38, 61] and supported the efficacy of shorter PBM exposure time (20 seconds per tooth) in reducing pain after band application.

In the present study, participants in the control group did not show the typical pain cycle characterized by a peak at 18-24 h after force application. However, band insertion is an orthodontic procedure causing minimal tooth movements [43] and probably a consequently different pain cycle and intensity as compared to other procedures such as separators or archwire placement.

Lastly, it is worth noting that prior evidence reported that patients' pain sensations might depend on individual characteristics such as age and gender that affect the pain threshold [3, 62] and analgesic treatment outcome. However, prior evidence, investigating pain perception, failed to report any difference due to age and gender [48, 63, 64]. Nevertheless, our data do not allow us to draw any definitive conclusion regarding the influence of age and gender on PBM efficacy for which future studies might be valuable.

The limitation of the present study might be that a single-blind design was adopted. Additionally, one single laser power output has been tested. Generalizability of the present findings might be limited by the fact that pain intensity was measured after band application on upper first molars only; therefore, further studies adopting the same PBM protocol on different teeth are necessary. Moreover, the recruitment of younger or older patients might help understand whether PBM effects are generalizable to patients with different ages.

In conclusion, the present study demonstrated the efficacy of PBM in decreasing pain intensity, especially in the first 12 hours after upper first molar band application. However, further double-blind studies with greater sample size and adopting objective pain measures are necessary to better define specific parameters in order to recommend PBM as a routine method for orthodontic pain control.

This trial was not registered.

The protocol was not published before trial commencement.

## Figures and Tables

**Table 1:** Baseline characteristics of patients in each group.

Demographic characteristics			
	Total sample	Trial group	Control group
*N*	26	13	13
Age (mean, SD)	11.8 (3.6)	11.9 (3.7)	11.7 (3.7)
Male (%)	34.6	30.7	38.5
Female (%)	65.4	69.3	61.5

Clinical characteristics			
	Mean (SD)	Mean (SD)	Mean (SD)
Oral plaque index	0.74 (0.21)	0.77 (0.18)	0.71 (0.24)
Gingival index 16	0.31 (0.32)	0.22 (0.30)	0.40 (0.33)
Gingival index 26	0.28 (0.31)	0.19 (0.27)	0.36 (0.33)
Probing pocket depth 16	2.22 (0.43)	2.09 (0.40)	2.35 (0.43)
Probing pocket depth 26	2.20 (0.31)	2.19 (0.25)	2.21 (0.37)
Clinical attachment level 16	0	0	0
Clinical attachment level 26	0	0	0

**Table 2:** Laser parameters.

Parameters	Values
Wavelength (nm)	830 ± 10
Spot of the probe (cm^2^)	0.1
Power output (mW)	150 ± 10
Exposure time in each point (s)	5
Energy density for each point (J/cm^2^)	7.5
Irradiation points for each tooth	4 (MB, DB, MP, DP)
Energy density in each tooth (J/cm^2^)	30
Application technique	In contact
Number of sessions	1
Operation mode	Continuous wave

**Table 3:** Descriptive statistics (WBS) of the two groups. ^∗^Statistical significance. Means with the same letters are not significantly different.

Group	Time	Mean	SD	Min	Mdn	Max	Significance^∗^
Control	T0	1.62	1.26	0.00	2.00	4.00	A
Control	T1	1.77	1.92	0.00	2.00	5.00	A
Control	T2	1.77	2.17	0.00	0.00	6.00	A
Control	T3	1.08	1.75	0.00	0.00	6.00	A, C, D
Control	T4	0.15	0.55	0.00	0.00	2.00	D
Trial	T0	0.92	1.32	0.00	0.00	4.00	C
Trial	T1	0.77	1.01	0.00	0.00	2.00	C
Trial	T2	0.77	1.54	0.00	0.00	4.00	C
Trial	T3	0.62	1.71	0.00	0.00	6.00	C, D
Trial	T4	0.31	0.75	0.00	0.00	2.00	D

**Table 4:** Results of the start, peak, and end of pain in hours.

Code	Question	Group	Mean	SD	Min	Mdn	Max	Significance
Q1	How many hours after the intervention did the pain start?	Control	5.25	7.86	0.00	1.00	24.00	ns
		Trial	10.86	26.97	0.00	1.00	72.00	

Q2	When did you have the most serious pain?	Control	6.17	7.96	0.00	1.00	24.00	ns
		Trial	15.86	26.29	0.00	1.00	72.00	

Q3	When did the pain disappear?	Control	22.08	25.42	0.00	18.00	72.00	ns
		Trial	39.57	31.33	1.00	24.00	72.00	

**Table 5:** Response frequencies of Q4 for both groups.

Group	No pain	Compressive pain from the appliance	Pain when biting firmly but without eating problems	Pain when eating	Spontaneous pain or pain which prevents eating	Total	Significance
Trial	6 (46.2%)	5 (38.5%)	1 (7.7%)	1 (7.7%)	0 (0%)	13 (100%)	*p* < 0.05
Control	1 (7.7%)	7 (58.8%)	1 (7.7%)	3 (23%)	1 (7.7%)	13 (100%)	

## Data Availability

All data are available upon request to the corresponding author.
